# Algorithmic and sensor-based research on Chinese children’s and adolescents’ screen use behavior and light environment

**DOI:** 10.3389/fpubh.2024.1352759

**Published:** 2024-02-22

**Authors:** Jifang Wang, Yang Shen, Jing Zhao, Xiaoying Wang, Zhi Chen, Tian Han, Yangyi Huang, Yuliang Wang, Wuxiao Zhao, Wen Wen, Xingtao Zhou, Ye Xu

**Affiliations:** ^1^Eye Institute and Department of Ophthalmology, Eye & ENT Hospital, Fudan University, Shanghai, China; ^2^NHC Key Laboratory of Myopia (Fudan University), Key Laboratory of Myopia, Chinese Academy of Medical Sciences, Shanghai, China; ^3^Shanghai Research Center of Ophthalmology and Optometry, Shanghai, China; ^4^Shanghai Engineering Research Center of Laser and Autostereoscopic 3D for Vision Care, Shanghai, China; ^5^Department of Nursing, Eye & ENT Hospital, Fudan University, Shanghai, China; ^6^Center for Optometry and Visual Science, Guangxi Academy of Medical Sciences, Nanning, China

**Keywords:** artificial intelligence, display terminal, light environment, myopia, screen time, viewing distance

## Abstract

**Background:**

Myopia poses a global health concern and is influenced by both genetic and environmental factors. The incidence of myopia tends to increase during infectious outbreaks, such as the COVID-19 pandemic. This study examined the screen-time behaviors among Chinese children and adolescents and investigated the efficacy of artificial intelligence (AI)-based alerts in modifying screen-time practices.

**Methods:**

A cross-sectional analysis was performed using data from 6,716 children and adolescents with AI-enhanced tablets that monitored and recorded their behavior and environmental light during screen time.

**Results:**

The median daily screen time of all participants was 58.82 min. Among all age groups, elementary-school students had the longest median daily screen time, which was 87.25 min and exceeded 4 h per week. Children younger than 2 years engaged with tablets for a median of 41.84 min per day. Learning accounted for 54.88% of participants’ screen time, and 51.03% (3,390/6,643) of the participants used tablets for 1 h at an average distance <50 cm. The distance and posture alarms were triggered 807,355 and 509,199 times, respectively. In the study, 70.65% of the participants used the tablet under an illuminance of <300 lux during the day and 61.11% under an illuminance of <100 lux at night. The ambient light of 85.19% of the participants exceeded 4,000 K color temperature during night. Most incorrect viewing habits (65.49% in viewing distance; 86.48% in viewing posture) were rectified swiftly following AI notifications (all *p* < 0.05).

**Conclusion:**

Young children are increasingly using digital screens, with school-age children and adolescents showing longer screen time than preschoolers. The study highlighted inadequate lighting conditions during screen use. AI alerts proved effective in prompting users to correct their screen-related behavior promptly.

## 1 Introduction

In recent decades, the global electronics industry has developed rapidly, accelerating the advent of the digital information age. Various digital products, including desktop computers, laptops, digital televisions, and smartphones, affect nearly all aspects of people’s lives. For instance, traditional forms of work, learning, social communication, and entertainment are being replaced by telecommuting, remote courses, cyber communication, and online entertainment, respectively. The use of digital devices is efficient and convenient; however, it prolongs near work and screen time, leading to video display terminal syndrome (or computer vision syndrome) (1–3).

The COVID-19 pandemic led to widespread school closures and in-house quarantines, which popularized remote education, telecommuting, and online entertainment. Unfortunately, this has also led to an increase in myopia worldwide (4–6). Studies have shown that school-aged children experienced a myopic refractive shift during the pandemic compared to the period before (7). Furthermore, myopia progression has accelerated in terms of spherical equivalent refractive (−0.83D) and axial length (0.36 mm) (8). Various environmental factors, such as increased digital screen time, near work, and limited outdoor and dim-light exposure, are considered significant risks of myopia onset and progression in children and adolescents, except for genetic factors (9, 10). Moreover, using digital screens at a short distance, with poor posture, under poor illumination, or improper color temperature increases the incidence of myopia (11, 12), strabismus (13), anisometropia (2), postural scoliosis (14), and abnormalities in musculoskeletal development among youth (15). However, children and adolescents often use electronic devices in an unsupervised environment, and thus, little is known about their screen-time behaviors, such as daily usage schedules, duration, frequency, viewing content, viewing posture, and viewing distance, and the ambient light environments, such as illuminance and color temperature.

This study employed commercially available artificial intelligence (AI)-enhanced tablets to map screen-time behaviors and luminous environments in Chinese children and adolescents during the COVID-19 pandemic. Furthermore, this study investigated the efficacy of AI-based visual and audible alerts in screen-time behavior correction among children and adolescents.

## 2 Methods

### 2.1 Participants and ethical approval

The study was conducted following the principles of the Declaration of Helsinki. Informed consent was obtained from all participants and their legal guardians, who agreed to disclose the identifying information in an online open-access publication. The study protocol was approved by the Ethics Committee of the Fudan University Eye and ENT Hospital Review Board (No. 2022113).

In the cross-sectional study, participants were users of commercially available AI-enhanced tablets (manufactured by BOE Yiyun Technology Co., Ltd., Changsha, Hunan Province, China; Model A127CS 6 + 128G) between October 9, 2022 and December 31, 2022. Participants aged over 18 years were excluded. Anonymous electronic survey questionnaires were sent to the participants and their legal guardians to verify the valid use of the tablets and collect information on the age and gender of the recruited children and adolescents. The tablets continuously recorded behavioral characteristics, including screen-time duration, frequency, content, viewing distance, body posture, head position, eye position, blinking frequency, and parameters of luminous environments (illumination and color temperature) during their use from October 9, 2022, to December 31, 2022. Data was collected from 6,716 children and adolescents (3,526 boys and 3,190 girls). Following the elimination of participants with missing data, data from 6,643 children and adolescents (3,485 boys and 3,158 girls, mean age 6.1 ± 3.0 years, 0–18 years old) were collected from 25 provinces and 4 municipalities across mainland China. No participants were infected during this period, except for those in Shanghai (*n* = 123) who were infected within their households. This was attributed to the liberalization of epidemic control measures in December 2022. Participants were divided into five groups according to age: participants aged from 0 to 2 years (before preschool) were enrolled in Group BPS, 3 to 5 years (preschool) in Group PS, 6 to 11 years (elementary school) in Group ES, 12 to 14 years (junior high school) in Group JHS, and 15 to 18 years (senior high school) in Group SHS.

### 2.2 Equipment

**Figure 1:**
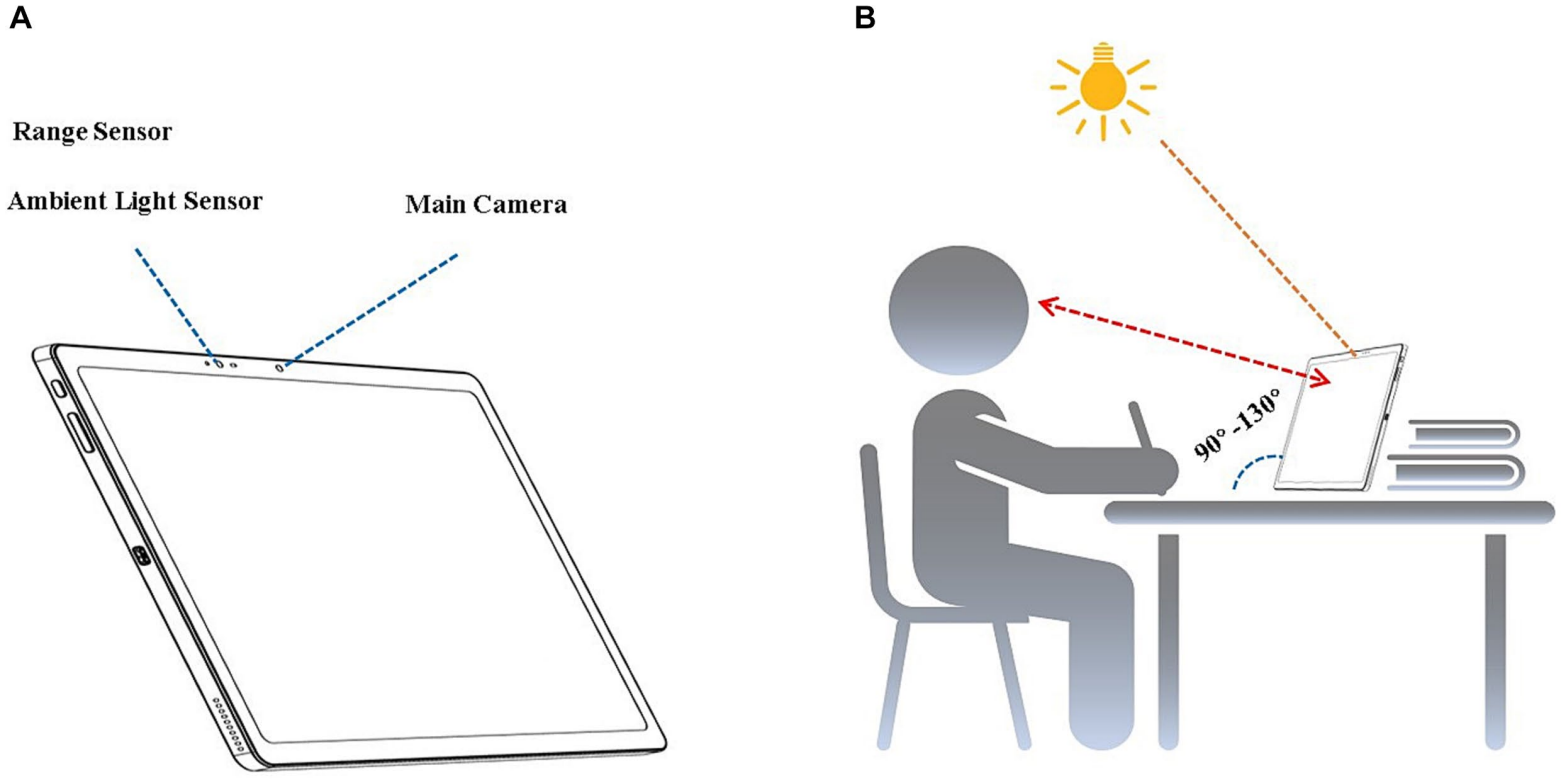
Equipment and measurements. **(A)** Artificial intelligence-enhanced tablet computer. **(B)** Viewing distance, posture, ambient illumination, and color temperature monitoring.

The tablets were 279 × 215.4 × 7.2 mm in size equipped with a capacitive touch screen (12.7 in) for display and interaction, main front digital camera (16 megapixels, G127, DMEGC) for photography and AI action recognition, secondary front digital camera (5 megapixels, G127, DMEGC) for posture monitoring, rear digital camera (13 megapixels, CL3A872 V2, C&T), flash, range sensor (ranging from 1 to 250 cm, TMF 8805, ams), and ambient light sensor (TCS3707, ams, Figure 1A). For privacy protection, only the outlines of the body, head and eyes were extracted and analyzed.

### 2.3 Screen-time duration and period

After each user logged into their account, the tablet usage time was accumulated when the camera obtained a stable image of their facial outline. The average daily and weekly tablet usage times were calculated using Eqs 1, 2:


(1) $$\begin{array}{l} \mathrm{Average}\;\mathrm{daily}\;\mathrm{usage}\;\mathrm{time}=\frac{\overset{n}{\underset{d=1}{\sum }}Xd}{n}\;(d:\mathrm{the}\;\mathrm{accumulated}\\ \mathrm{usage}\;\mathrm{in}\;\mathrm{one}\;\mathrm{day};\mathrm{only}\;\mathrm{usage}\;\mathrm{times}\;\mathrm{longer}\;\mathrm{than}\;5\;\min\;\\ \mathrm{per}\;\mathrm{time}\;\mathrm{were}\;\mathrm{included}) \end{array}$$

(2) $$\begin{array}{l} \mathrm{Average}\;\mathrm{weekly}\;\mathrm{usage}\;\mathrm{duration}=\frac{\overset{n}{\underset{w=1}{\sum }}Xw}{n}\;\\ (w:\mathrm{the}\;\mathrm{accumulated}\;\mathrm{usage}\;\mathrm{time}\;\mathrm{in}\;\mathrm{one}\;\mathrm{week};\\ \mathrm{only}\;\mathrm{usage}\;\mathrm{times}\;\mathrm{collected}\;for\;\mathrm{seven}\;\mathrm{consecutive}\\ \mathrm{days}\;\mathrm{were}\;\mathrm{included}) \end{array}$$

Sixty-eight applications (apps) with the most usage during the study period were classified into social networking, search engines, entertainment, and education apps. The duration of use (accumulated usage time) and time intervals (morning, 6:00–11:00; noon, 11:00–13:00; afternoon, 13:00–16:00; evening, 16:00–18:00; night, 18:00–24:00; and before dawn, 0:00–6:00) were recorded for each category.

### 2.4 Ambient illumination and color temperature

The ambient light sensor measured the ambient illumination and color temperature during screen time. The ambient illuminance values obtained during three-time intervals (daytime, 8:00 to 20:00; nighttime, 20:00 to 8:00; and the entire day) were, respectively, divided into four illumination intervals (rows): (0 lux, 100 lux), [100 lux, 300 lux), [300 lux, 1,000 lux), [1,000 lux, 4,000 lux]. In each time interval, the exposure time ratio of each illuminance interval was calculated as.


$$\frac{\mathrm{Exposure}\;\mathrm{duration}\;\mathrm{under}\;a\;\mathrm{certain}\;\mathrm{illuminance}\;\mathrm{interval}}{\mathrm{Duration}\;\mathrm{of}\;\mathrm{the}\;\mathrm{time}\;\mathrm{interval}}\times 100\%$$

The color temperature values (kelvin, K) obtained during the three-time intervals were divided into four intervals: (0 K, 4,000 K), [4,000 K, 6,000 K), [6,000 K, 8,000 K), and [8,000 K, +∞ K). In each time interval, the exposure time ratio of each color temperature interval was calculated as.


$$\frac{\begin{array}{l} \;\mathrm{Exposure}\;\mathrm{duration}\;\mathrm{under}\;a\;\mathrm{certain}\;\mathrm{color}\\ \;\mathrm{temperature}\;\mathrm{interval} \end{array}}{\mathrm{Duration}\;\mathrm{of}\;\mathrm{the}\;\mathrm{time}\;\mathrm{interval}}\times 100\%$$

### 2.5 Viewing distance

The viewing distance was measured using a range sensor combined with the tablet’s main front camera. After the main camera captured the facial outline, the range sensor obtained the distance between the screen and face plane every 3 s. The viewing-distance data were considered valid only when the angle between the screen and desktop was 90° to 130° (Figure 1B). The average viewing distance was calculated and analyzed. When the viewing distance was detected to be less than 30 cm for 12 s, and an audio alert was played for 10 s to remind users to maintain a sufficient distance during screen time. If the user corrected the viewing distance to beyond 30 cm in 10 s, the alert was stopped and recorded as effective. If the user ignored the alert, a pop-up notification appeared on the screen, interrupting the user’s interaction with the tablet until the user adjusted the viewing distance to beyond 35 cm. The audio and pop-up notifications were recorded, and the effective alert ratio was calculated.

### 2.6 Blink frequency

Eye blinks, lasting between 50 to 500 ms (16), represent brief eyelid closures. Blink frequency, measured as the number blinks per minute, was assessed using the primary front camera. This camera continuously observed users’ pupil and eyelid movements, with AI models detecting blink occurrences. Blink actions that persisted for over 1 min were documented.

### 2.7 Identification of viewing posture and alerting

**Figure 2:**
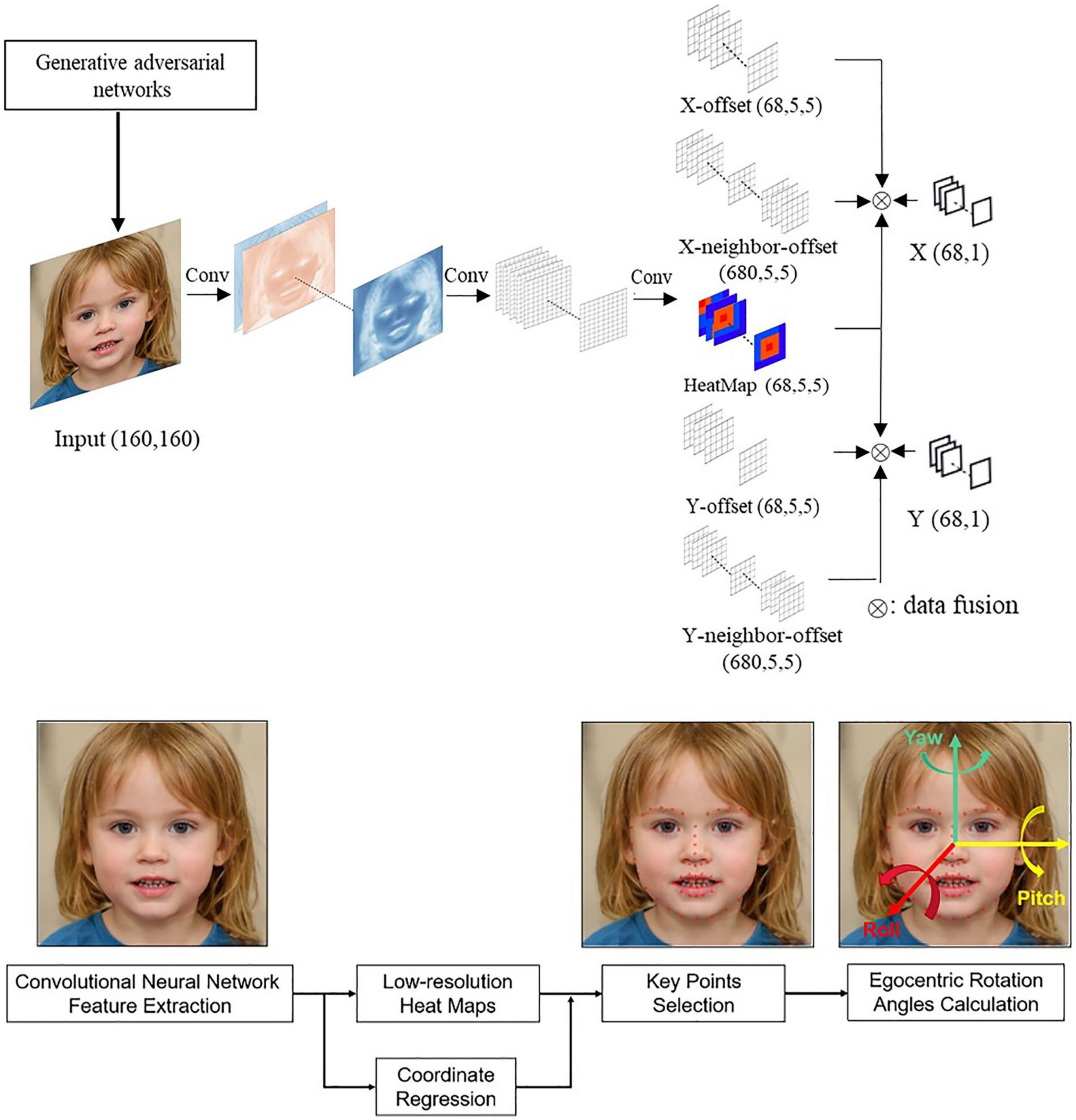
Artificial intelligence model: Identify and Alert poor postures and improper viewing distance. The top flowchart is the generative adversarial network to coordinate the offset of the X and Y dimensions. The bottom flowchart is the convolutional neural network to compute egocentric rotation angles.

Head posture refers to the position and orientation of the three-dimensional space of the head. Head postures are described by the egocentric rotation angles of pitch, roll, and yaw (17). Using a low-resolution thermal map regression model, semi-supervised learning with a self-training algorithm was employed. To increase the robustness of the model, generative adversarial networks (GANs) were used to generate facial data for different ages, skin colors, and angles. A total of 300,000 images (100,000 labeled and 200,000 unlabeled) were included in the training set and 10,000 images in the testing set (Figure 2).

In this study, the head was considered to be tilted when the roll angle exceeded ±30° for more than 12 s. When a tilted head was detected, an audio alert reminded the user to correct their head posture within the next 10 s. If the user ignored the alert, a pop-up notification appeared on the screen to interrupt the tablet use until the user corrected their head pose. The total number of audio alerts and pop-up notifications were recorded, and the effective alert ratio was calculated.

### 2.8 Statistical analysis

Statistical analyses were performed using SPSS version 20.0. Data were presented as mean ± standard deviation. Mixed-effects models were used to analyze differences in usage time and viewing distance, with age brackets as the intra-group factors, as well as the blink frequency, changed over time. R × C chi-square tests were also employed to compare the difference in the effective alert ratios of viewing distance and head posture among age brackets.

## 3 Results

### 3.1 Demographics

Among the 6,643 Chinese children and adolescents recruited for this study, Groups BPS, PS, ES, JHS, and SHS included 607, 2,370, 3,370, 204, and 92 participants, respectively. The Gross Domestic Product (GDP) values corresponding to the geographical areas where these participants were located, as well as the average age and gender of participants from each area, are listed in Table 1.

**Table 1:** Demographics (Mean ± SD).

Province/City	Sample size	Gender	Age	*Per Capita* GDP in 2022	Daily tablet usage time
		Boys: Girls	(years)	(RMB yuan)	(Minutes)
Beijing	1,392	719:673	6.2 ± 3.0	190,091	69 ± 49
Shanghai	367	189:178	5.6 ± 2.6	179,401	59 ± 43
Yunnan	61	36:25	6.2 ± 3.1	61,736	58 ± 47
Inner Mongolia	112	67:45	5.8 ± 2.8	96,496	58 ± 39
Jilin	150	91:59	6.1 ± 2.9	55,033	70 ± 50
Sichuan	246	122:124	5.8 ± 2.7	67,785	48 ± 37
Tianjin	265	148:117	6.2 ± 2.8	118,801	67 ± 51
Ningxia	15	8:7	6.2 ± 2.3	69,925	76 ± 59
Anhui	151	70:81	5.7 ± 3.0	73,687	45 ± 33
Shandong	359	196:163	5.9 ± 3.3	85,793	61 ± 47
Shanxi	171	83:88	6.3 ± 2.8	73,686	66 ± 49
Guangdong	429	213:216	6.2 ± 3.0	101,796	51 ± 41
Guangxi	33	21:12	6.7 ± 3.9	52,215	44 ± 32
Xinjiang	32	20:12	4.8 ± 2.3	68,526	52 ± 47
Jiangsu	405	224:181	5.6 ± 3.0	144,475	44 ± 35
Jiangxi	85	38:47	6.1 ± 2.5	71,009	45 ± 26
Hebei	252	135:117	6.4 ± 3.1	56,888	72 ± 51
Henan	199	106:93	6.2 ± 3.1	62,071	63 ± 44
Zhejiang	320	162:158	5.8 ± 3.0	118,830	40 ± 35
Hainan	63	38:25	6.0 ± 3.3	66,845	56 ± 39
Hubei	315	168:147	6.8 ± 3.1	92,170	83 ± 60
Hunan	94	47:47	5.8 ± 2.8	73,498	48 ± 35
Gansu	35	17:18	5.9 ± 2.5	44,986	65 ± 47
Fujian	146	79:76	5.7 ± 2.8	126,845	45 ± 34
Guizhou	30	17:13	5.8 ± 2.5	52,348	28 ± 15
Liaoning	404	218:186	6.2 ± 2.9	68,515	65 ± 49
Chongqing	140	74:66	5.5 ± 3.0	90,688	67 ± 50
Shaanxi	175	85:90	6.1 ± 3.1	82,885	48 ± 31

GDP, gross domestic product.

### 3.2 Usage time

The median daily usage time and sample sizes of each group are listed in Table 2. A significant difference in daily usage time was observed among the five groups (*F* = 255.72, *p* < 0.001). Bonferroni *post hoc* tests showed that the daily usage time of Group ES was significantly longer than that of the other groups (all *post hoc p* < 0.05); the daily usage times of Groups JHS and SHS were the second longest (*post hoc p* = 0.354) and longer than those of Groups BPS and PS (all *post hoc p* < 0.001). The daily usage times of Groups BPS and PS were similar (*post hoc p* = 1.000).

**Table 2:** Daily usage time of each group with median (P25, P75).

Age groups	Sample size	Daily usage time (minutes)	*F* ^a^	*p* ^b^	*Post hoc p* ^c^
BPS (0–2 y)	607	41.84 (23.27, 75.28)			BPS vs. PS, *p* = 1.000
					BPS vs. ES, *p* < 0.001^*^
PS (3–5 y)	2,370	45.36 (26.99, 73.81)			BPS vs. JHS, *p* < 0.001^*^
					BPS vs. SHS, *p* < 0.001^*^
ES (6–11 y)	3,370	87.25 (42.89,136.34)	225.72	<0.001	PS vs. ES, *p* < 0.001^*^
					PS vs. JHS, *p* < 0.001^*^
JHS (12–14 y)	204	60.26 (28.47, 132.03)			PS vs. SHS, *p* < 0.001^*^
					ES vs. JHS, *p* = 0.022^*^
SHS (15–18 y)	92	53.02 (24.81, 109.48)			ES vs. SHS, *p* = 0.004^*^
					JHS vs. SHS, *p* = 0.354

BPS, before preschool; PS, preschool; ES, elementary school; JHS, junior high school; SHS, senior high school; yrs, years; a, b = Mixed-effects model; c = Bonferroni post hoc tests; ^*^ = statistically significant.

The weekly usage time of all the groups is listed in Table 3. The median weekly usage time of Groups ES (562.03 min), JHS (566.73 min), and SHS (425.35 min) were significantly longer than those of Groups BPS (all *post hoc p* ≤ 0.001) and PS (all *post hoc* p < 0.001). However, no significant differences were detected in the weekly usage time between Groups BPS and PS (*post hoc* p = 1.000) and Groups ES, JHS, and SHS (all *post hoc p* > 0.05).

**Table 3:** Weekly usage time of each group with median (P25, P75).

Age groups	Sample size	Weekly usage time (minutes)	*F* ^a^	*p* ^b^	*Post hoc p* ^c^
BPS (0–2 y)	607	151.85 (70.15, 287.28)			BPS vs. PS, *p* = 1.000
					BPS vs. ES, *p* < 0.001^*^
PS (3–5 y)	2,370	166.28 (86.46, 293.94)			BPS vs. JHS, *p* < 0.001^*^
					BPS vs. SHS, *p* = 0.001^*^
ES (6–11 y)	3,370	315.86 (143.50, 562.03)	198.96	<0.001	PS vs. ES, *p* < 0.001^*^
					PS vs. JHS, *p* < 0.001^*^
JHS (12–14 y)	204	198.33 (91.47, 566.73)			PS vs. SHS, *p* < 0.001^*^
					ES vs. JHS, p = 1.000
SHS (15–18 y)	92	181.24 (60.95, 425.35)			ES vs. SHS, *p* = 0.200
					JHS vs. SHS, *p* = 0.288

BPS, before preschool; PS, preschool; ES, elementary school; JHS, junior high school; SHS, senior high school; yrs, years; a, b = Mixed-effects model; c = Bonferroni post hoc tests; * = statistically significant.

### 3.3 Luminance and ambient lighting color temperature

**Figure 3:**
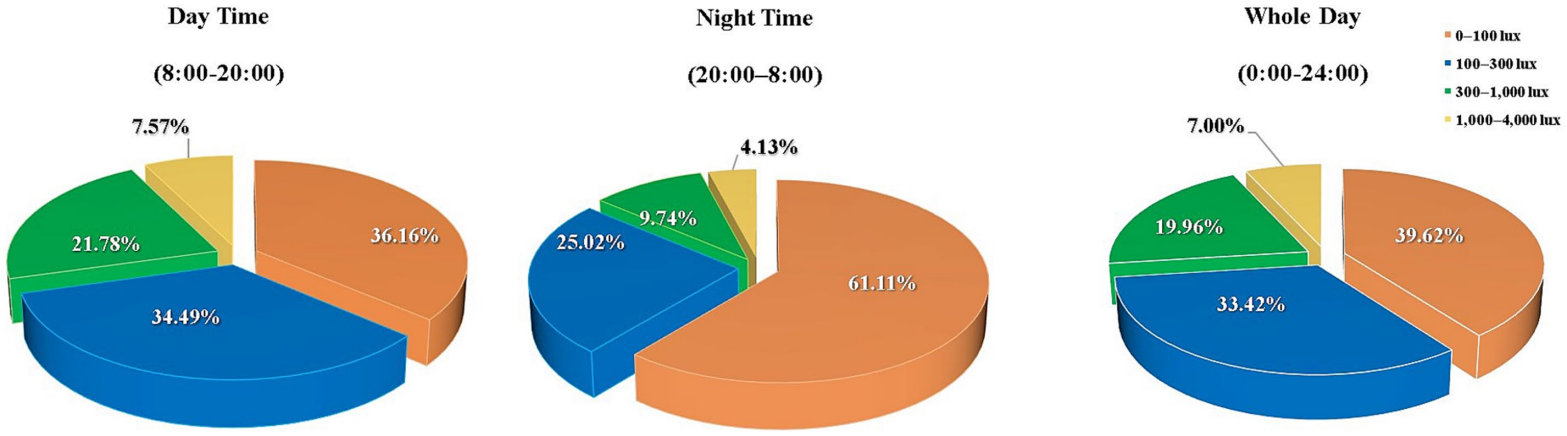
Distribution of luminance during screen time. Lux, luminance.

**Figure 4:**
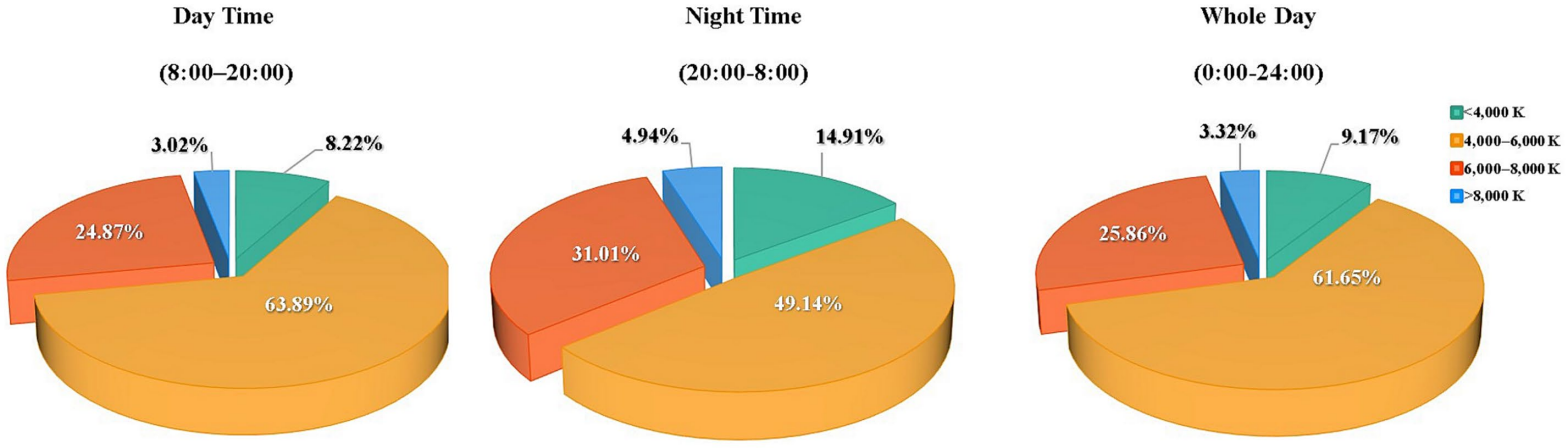
Distribution of ambient lighting color temperature during screen time. K, kelvin.

Figure 3 lists the findings of the tablet computer’s ambient light sensor, indicating that during the day, luminance levels fell below 100 lux for 36.16%, between 100–300 lux at 34.49%, 300–1,000 lux at 21.78% and above 1,000 lux at 7.57% of the screen time. During the night, these levels fell below 100 lux at 61.11%, between 100–300 lux at 25.02%, 300–1,000 lux at 9.74% and above 1,000 lux at 4.13% of the screen time. Figure 4 details the distribution of ambient lighting color temperature during screen time. In daytime conditions, the color temperature was below 4,000 K at 8.22%, between 4,000- 6000 K at 63.89%, 6,000-8000 K at 24.87% and above 8,000 K at 3.02% of the screen time. During nighttime usage, it was below 4,000 K at 14.91%, between 4,000-6000 K at 49.14%, 6,000-8000 K at 31.01% and above 8,000 K at 4.94% of the screen time.

### 3.4 Viewing content

**Figure 5:**
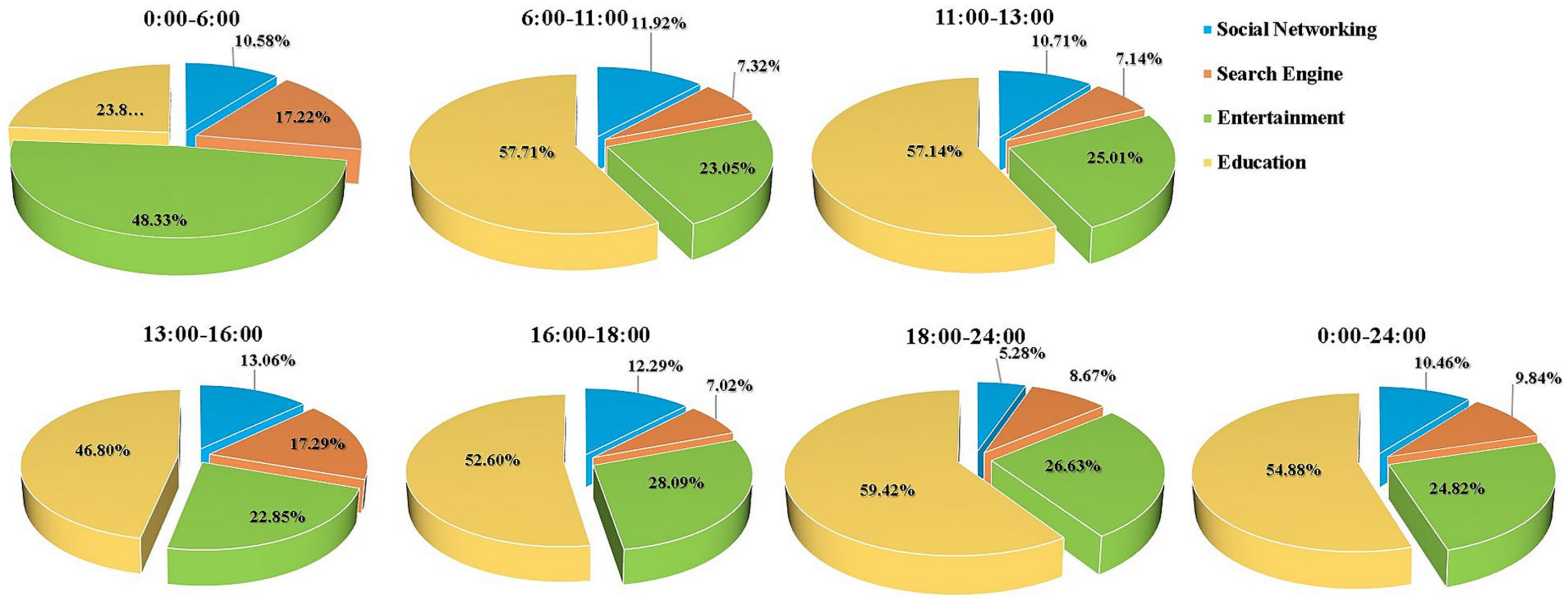
View content at different times of the day (social networking, search engine, entertainment, and education).

The top 48 most used applications (apps) were filtered from 5,424 apps and classified into 4 categories: social networking, search engines, entertainment, and education. Their session durations were recorded (Figure 5). Educational applications accounted for 54.88% (3,646/6,643) of the sessions over the entire day (ranked first in all categories), including the most significant proportion of all session durations except for the before-dawn period, in which entertainment applications ranked first (48.33%; 478/988).

### 3.5 Blinking rate

Table 4 presents the blinking rates observed across six-time slices for each age group.

**Table 4:** Blinking rate (Mean ± SD).

Time Slice/Age Group	BPS(*N* = 232)	PS(*N* = 952)	ES(*N* = 1814)	JHS(*N* = 100)	SHS(*N* = 37)	*F* ^a^	*p* ^b^
0–10 min (t/m)	10.94 ± 5.89	11.81 ± 5.67	14.32 ± 6.28	14.60 ± 6.76	13.80 ± 6.67	38.502	< 0.001
10–20 min (t/m)	10.08 ± 5.65	11.41 ± 6.18	14.49 ± 6.78	14.20 ± 7.12	15.09 ± 8.12	54.388	< 0.001
20–30 min (t/m)	10.48 ± 6.47	11.38 ± 6.52	14.29 ± 7.33	15.77 ± 10.26	14.27 ± 7.21	39.553	< 0.001
30–40 min (t/m)	10.67 ± 7.52	11.36 ± 7.24	14.14 ± 7.48	14.60 ± 7.84	14.62 ± 9.04	29.567	< 0.001
40–50 min (t/m)	10.43 ± 7.93	11.10 ± 7.58	14.41 ± 8.09	14.10 ± 8.28	13.69 ± 8.28	35.385	< 0.001
50–60 min (t/m)	10.36 ± 8.30	11.37 ± 8.49	14.25 ± 7.49	14.91 ± 9.38	14.37 ± 9.02	25.138	< 0.001

BPS, before preschool (0–2 yrs); PS, preschool (3–5 yrs); ES = elementary school (6–11 yrs); JHS = junior high school (12–14 yrs); SHS = senior high school (15–18 yrs); yrs = years; N = number of sample size; min = minutes; t/m = times per minute; a, b = Mixed-effects model.

These rates exhibited minor fluctuations over time for each age group (*p* > 0.05). However, significant variations were noted among age groups during the following time slices: 0–10 min (*F* = 38.502, *p* < 0.001), 10–20 min (*F* = 54.388, *p* < 0.001), 20–30 min (*F* = 39.553, *p* < 0.001), 30–40 min (*F* = 29.567, *p* < 0.001), 40–50 min (*F* = 35.385, *p* < 0.001), and 50–60 min (*F* = 25.138, *p* < 0.001). Bonferroni *post hoc* comparisons revealed that the blinking rates of Groups ES and JHS were significantly higher than those of Groups BPS and PS in all six-time slices (all *post hoc p* < 0.01). However, no significant difference in blinking rates were observed between Groups BPS and PS (all *post hoc p* > 0.05) or among Groups ES, JHS, and SHS (all *post hoc p* > 0.05) in all-time slices.

### 3.6 Efficacy of AI models in viewing distance and posture alert

Tables 5, 6 show that 6,378 and 6,403 participants used viewing distance and posture monitoring, respectively. Of the subjects, 83.76% (5,564/6,643) used the tablets with an average viewing distance of less than 50 cm, 60.71% (4,033/6,643) used the tablets continuously for 1 h, and 51.03% (3,390/6,643) used the tablets continuously for 1 h with a viewing distance of less than 50 cm. In total, 807,355 and 509,199 improper viewing distance and posture alerts were triggered, respectively. The ES group had the highest per-capita alerts (479.58) of viewing distance among all groups. The effects of viewing-distance correction among the groups differed significantly (*χ*^2^ = 1,174.165, *p* < 0.001). The efficacy of viewing-distance correction in the JHS group (324,269/479,567; 69.67%) was the highest of all groups (all *post hoc p* < 0.05), whereas that in Group BPS (25,442/41,409; 61.44%) was the lowest (all *post hoc p* < 0.05). The per-capita posture alerts were the highest in Group BPS (90.58). The efficacy of the AI alerts in posture correction differed among the groups (*χ*^2^ = 2,519.789, *p* < 0.001). The effectiveness of the posture correction in Groups ES (235,254/267,951; 87.80%) and SHS (5,238/5,984, 87.62%) were significantly higher than those in the other groups (all *post hoc p* < 0.05). The difference in efficacy between Groups ES and SHS was non-significant (*post hoc p* > 0.05).

**Table 5:** Efficacy of alerts in viewing-distance correction.

Age groups	Sample size	Total alert (times)	*Per Capita* alert (times)	Valid alert (times)	Invalid alert (times)	Alert efficacy (%)	χ^2^	*p*
BPS	562	41,409	73.68	25,442	15,967	61.44^a^	1174.165	<0.001
PS	2,283	254,870	111.64	164,947	89,923	64.72^b^		
ES	3,259	479,567	479.58	324,269	155,298	67.62^c^		
JHS	188	15,604	83.00	10,871	4,733	69.67^d^		
SHS	86	15,905	184.94	10,485	5,420	65.92^e^		
Total	6,378	807,355	126.58	536,014	271,341	66.39		

BPS, before preschool (0–2 yrs); PS, preschool (3–5 yrs); ES, elementary school (6–11 yrs); JHS, junior high school (12–14 yrs); SHS, senior high school (15–18 yrs); yrs = years; Alert Efficacy = Valid Alert/ Total Alert×100%; a,b,c,d,e = Bonferroni post hoc tests (Pairwise comparisons between age groups were statistically significant, all post hoc *p* < 0.05).

**Table 6:** Efficacy of Artificial intelligence alerts in posture correction.

Age groups	Sample size	Total alert (times)	*Per Capita* alert (times)	Valid alert (times)	Invalid alert (times)	Alert efficacy	χ^2^	*p*
BPS	587	53,172	90.58	42,784	10,388	80.46^a^	2519.789	<0.001
PS	2,314	169,150	73.09	142,326	26,824	84.14^b^		
ES	3,223	267,951	83.14	235,254	32,697	87.80^c^		
JHS	195	12,942	66.37	10,952	1,990	84.62^b^		
SHS	84	5,984	71.24	5,238	746	87.53^c^		
Total	6,403	509,199	79.53	436,554	72,645	85.73		

BPS, before preschool (0–2 yrs); PS, preschool (3–5 yrs); ES, elementary school (6–11 yrs); JHS, junior high school (12–14 yrs); SHS, senior high school (15–18 yrs); yrs, years; Alert Efficacy = Valid Alert/ Total Alert×100%; a = Bonferroni post hoc tests (Pairwise comparisons between the BPS Group and other groups were statistically significant, *p* < 0.05), b = Bonferroni post hoc tests (The difference between group PS and JHS was nonsignificant, *p* > 0.05), c = Bonferroni post hoc tests (The difference between group ES and SHS was nonsignificant, *p* > 0.05).

## 4 Discussion

### 4.1 Emergence of electronic screen usage in younger children

During the COVID-19 pandemic, most educational institutions substituted online courses for in-person classes. Students studied at home using electronic devices. As most outdoor activities were restricted, leisure time was filled with online entertainment, and the duration of near work dramatically increased. This study found that 0–2-year-old children used tablets for more than 40 min per day on median, violating the recommendation of the American Academy of Pediatrics to avoid digital media use (except video chatting) in children younger than 18 to 24 months (18).

Moreover, excessive use of digital screens in early childhood may delay the development of cognition, language, and emotions (2). The median screen time for elementary-school students was more than 4 h (315.86 min) per week, and the median daily screen time approached 1.5 h (87.25 min), which is the longest among all age groups. This finding differs from the results of Wang et al.’s investigation (19). Saxena et al. (20) reported that children and adolescents who use electronic screens for more than 4 h per week are at more than eight times higher risk of contracting myopia than those who do not use electronic screens. A systematic review and meta-analysis indicated that the prolonged use of electronic screens could significantly increase the axial eye length and risk of myopia (21). Furthermore, excessive electronic screen time may replace outdoor activity time, reduce natural light exposure, increase the risk of obesity (22), and decrease sleep quality (23), increasing the risk of myopia occurrence and progression.

### 4.2 Prolonged use of electronic screens at close range by children and adolescents

Regarding the rhythm and viewing distance of tablet usage, our findings demonstrate that the vast majority of participants (83.76%; 5,564/6,643) had an average distance of less than 50 cm, and more than half of the participants (51.03%; 3,390/6,643) used the tablets continuously for 1 h at such viewing distances. The distance alarm was triggered 807,355 times. A study using apps to record the smartphone usage time of teenagers showed that in the group with fewer outdoor activities, the myopic diopter was lower for those who rested every 20 min after watching the screen (24). Additionally, prolonged use of electronic display devices at close distances can cause asthenopia. The incidence of dry eye syndrome in children has reached 50–60%, and its symptoms include dry eyes, foreign body sensation, tears, blurred vision, headache, esotropia, and abnormal vision. A study of 52 children in grades 3–4 revealed that the level of asthenopia was lowest when the screen was approximately 50 cm from the eyes (3). Reid et al. (25) proposed that children and adolescents follow the 20–20-20 principle while using digital products: rest and look at an object 20 feet away for 20 s after using the devices for 20 min. According to a recent study, it is recommended to rest and look further for more than 5 min to relieve asthenopia (26).

### 4.3 Increased blink frequency in school-aged children compared to preschoolers during screen use

Decreased blinking rates, increased tear evaporation, and aggravation of dry eye symptoms are significant contributors to asthenopia when individuals focus on screens. An investigation involving children in grades 3 and 4 revealed that blinking rates increased after an average of 15 min of tablet usage and were positively correlated with the duration of screen time (3). This study also identified notable differences in blinking rates among various age groups. Specifically, elementary, middle, and high school students, who spent more time on screens, exhibited significantly higher blinking rates than preschool and infant groups with shorter screen exposure. These findings align with those of Feng et al. (3), suggesting that the intensity of daily near work was greater for elementary, middle, and high school students. However, during the initial hour of screen time, the blinking rate in each age group fluctuated only slightly, with no statistically significant differences. This contrasts the results reported by Feng et al. (3). One possible explanation for this disparity is that our study’s baseline blinking rate, averaging 14 times per minute, was notably higher than the 8–10 times per minute reported by Feng et al. (3). Second, the blinking rate of each participant was measured in their homes, where they may have engaged in other near work activities, such as traditional reading and homework, prior to electronic screen use. Finally, variations in temperature, humidity, and illuminance conditions at home compared to laboratory settings may introduce confounding factors affecting blinking rates.

### 4.4 Children and adolescents use of electronic screens in low-illuminance environments

This study discovered that over 50% of the time was spent on learning, except during the pre-dawn period. This finding is consistent with those of Ma et al. (27) and Kohmarn et al. (28). In the present study, more than 70% of the children and adolescents used tablets with illuminance below 300 lux during the day, more than 85% of them used tablets with illuminance below 300 lux and more than 60% of them used tablets with illuminance below 100 lux at night. In addition, traditional paper-based work was often involved. These findings indicate insufficient home illumination, which is consistent with the results of cloud clip (29) monitoring of daily white light exposure (253 ± 36 lux) and light at eye level at night (139.6 ± 82.7 lux) (30). According to the dark-focus theory, the refractive state of the human eye will drift toward myopia in a dim-light environment, which necessitates engaging in proximate tasks such as reading and writing (31, 32). Furthermore, low illumination can easily cause a decrease in contrast on paper media, which reduces accommodation accuracy, causes blurred vision, aggravates asthenopia, and even induces myopia (33). In addition, an electronic screen is a self-luminous medium, and a low-ambient light and luminous display screen will lead to uneven space illumination and glare.

### 4.5 Children and adolescents use of electronic screens under ambient light with high color temperature at night

When monitoring the light environment with electronic screens, we found that for more than 85% of the participants, the ambient light exceeded 4,000 K color temperature at night, and it exceeded 6,000 K color temperature for more than 35% of the participants. Several studies (34, 35) have suggested that high-color temperature lighting at night disrupts the melatonin secretion cycle, influences sleep in children and adolescents, and adversely affects physical and psychological development. Sleep deprivation and poor sleep quality are risk factors for myopia occurrence and progression. In addition, the content displayed by the tablet computer inevitably contains high color temperature information, which can affect the hormone secretion rhythm in a specific period. We found that 1,612 participants used the tablets in the early morning (midnight to 6:00), whereas during lunch (11:00–13:00) and dinner (16:00–18:00), more than 6,000 of the total 6,643 participants were still recorded using electronic screens; however, the cumulative time was significantly lower than that in the morning, afternoon, and evening. The American Children’s Association recommends that children and adolescents avoid using electronic devices before and after meals and 1 h before sleep (18). The vast majority (more than 90%) of the participants in this study violated this recommendation. The content on electronic screens in the early morning was primarily focused on entertainment. This period is likely to be without parental supervision. Therefore, it is necessary to assist parents in managing the light environment, duration, and content on children’s and adolescents’ electronic screens.

### 4.6 Effective use of AI in correcting poor screen-use behaviors among children and adolescents

In this study, most children and adolescents received correction alerts for viewing distance (84.81%;5,409/6,378) and viewing posture (84.97%;5,441/6,403), respectively, while using tablets. This indicated that this demographic generally exhibits incorrect viewing behavior when using electronic screens. Notably, elementary school students received the highest number of alerts for viewing distance (479.58 times per person). This trend could be attributed to the substantial increase in learning tasks during this developmental stage, coupled with a potential lack of cognitive maturity, self-discipline, and healthy near work habits. Previous research (19) has found that the growth rate of myopia in children in lower-grade elementary schools during the COVID-19 period was the highest. This indicates that children aged 6–8 years may be especially responsive to environmental factors, heightened use of eyes in close proximity indoors, and decreased outdoor activities, all of which affect the onset and progression of myopia. This observation is also supported by Vander Veen et al. (36). In our study, most behaviors (65.49% for viewing distance and 86.48% for viewing posture) were corrected within 10 s after the AI alerts, with the highest correction rate in the middle school group (69.67%; 10,871/15,604) and the lowest correction rate in the before preschool group (61.44%; 164,947/254,870); this indicated that the application of AI can help monitor and correct unhealthy behaviors on electronic screen usage in children and adolescents; and older children and adolescents, such as middle school students, are able to respond more promptly and proactively under reminders to correcting bad viewing behaviors when using electronic screens, which might be correlated with their more complete cognitive and autonomous behavior control and correction abilities than younger preschool children. Therefore, it is recommended that parents provide more intensive supervision and guidance to younger children when they use electronic screens.

### 4.7 Practical implications

The findings of this study underscore the potential of AI technologies in assisting parents to monitor, warn, correct poor behaviors of near work and develop good viewing habits in children using electronic screens. These technologies have the capacity to facilitate the development of healthy viewing habits among children, thus helping to anticipate and mitigate the risk factors associated with children’s eye health. This may pave the way for novel approaches to myopia prevention and control, enabling parents to intervene proactively. As a result, there is a transition from clinical settings to the home environment in the prevention and management of myopia. Furthermore, this study lays the groundwork for the establishment of standards and guidelines on eye health care for children who use electronic screens. These guidelines encompass aspects such as viewing distance, view time, indoor ambient lighting illuminance, and color temperature in both home or classroom, for children and adolescents using electronic screens. Such standards contribute to the normalization of intelligent electronic screens for children and adolescents, fostering advancements in related industries.

### 4.8 Strengths and limitations

This study used the novel method of AI identification and monitoring to objectively measure the data of incorrect viewing behaviors and ambient light parameters during screen time among children and adolescents. And the inclusion of participants from over 20 provinces and municipalities ensured a diverse and geographically widespread representation of samples. However, the sample sizes extracted from different regions differed considerably, and the deviation between the sampling results and the population may be significant. Moreover, data were collected during the COVID-19 pandemic. The time, duration, and rhythm of children’s and adolescents’ screen time, like more online courses and longer continuous electronic screen usage because of more time at home, etc., differed from those during the normal period. After the relaxation of epidemic restrictions, 1.83% of the randomly selected sample were found to be infected. This may have led to a slight reduction in screen time for this group. Last, this study only monitored and investigated the screen-time behavior of children and adolescents during the use of BOE devices and did not monitor the participants’ use of other electronic devices, such as mobile phones, TVs, desktop computers, and tablets of other brands.

## 5 Conclusion

In summary, children of younger ages have started using electronic screens. Screen time for school-age children and adolescents with a closer viewing distance and higher incidence of poor viewing posture was longer than that for preschool children. During screen time, illumination was insufficient, paired with a high color temperature at night. AI can effectively monitor the time, content, and light environment during screen time and remind children and adolescents to correct their poor posture and behavior. Future research endeavors should consider adopting more rigorous sampling techniques, extended monitoring and observation periods, and the inclusion of supplementary online reporting for comprehensive data collection. This could involve gathering additional data on aspects such as time spent on other electronic devices, among other relevant factors.

## Data availability statement

The original contributions presented in the study are included in the article/supplementary material, further inquiries can be directed to the corresponding authors.

## Ethics statement

The studies involving humans were approved by the Ethics Committee of the Fudan University Eye and ENT Hospital Review Board. The studies were conducted in accordance with the local legislation and institutional requirements. Written informed consent for participation in this study was provided by the participants' legal guardians/next of kin.

## Author contributions

JW: Data curation, Formal analysis, Funding acquisition, Writing – original draft, Project administration. YS: Conceptualization, Funding acquisition, Writing – original draft. JZ: Formal analysis, Writing – original draft. XW: Conceptualization, Writing – review & editing. ZC: Funding acquisition, Writing – review & editing. TH: Formal analysis, Writing – original draft. YH: Formal analysis, Writing – original draft. YW: Data curation, Writing – original draft. WZ: Formal analysis, Funding acquisition, Writing – review & editing. WW: Formal analysis, Writing – review & editing. XZ: Funding acquisition, Supervision, Writing – review & editing. YX: Conceptualization, Supervision, Writing – review & editing.
